# Clinical Manifestations of Portal Hypertension

**DOI:** 10.1155/2012/203794

**Published:** 2012-09-17

**Authors:** Said A. Al-Busafi, Julia McNabb-Baltar, Amanda Farag, Nir Hilzenrat

**Affiliations:** ^1^Department of Medicine, College of Medicine and Health Sciences, Sultan Qaboos University, P.O. Box 35, 123 Muscat, Oman; ^2^Department of Gastroenterology, Royal Victoria Hospital, McGill University Health Center, Montreal, QC, Canada H3A 1A1; ^3^Department of Medicine, Royal Victoria Hospital, McGill University Health Center, Montreal, QC, Canada H3A 1A1; ^4^Department of Gastroenterology, Jewish General Hospital, McGill University, Montreal, QC, Canada

## Abstract

The portal hypertension is responsible for many of the manifestations of liver cirrhosis. Some of these complications are the direct consequences of portal hypertension, such as gastrointestinal bleeding from ruptured gastroesophageal varices and from portal hypertensive gastropathy and colopathy, ascites and hepatorenal syndrome, and hypersplenism. In other complications, portal hypertension plays a key role, although it is not the only pathophysiological factor in their development. These include spontaneous bacterial peritonitis, hepatic encephalopathy, cirrhotic cardiomyopathy, hepatopulmonary syndrome, and portopulmonary hypertension.

## 1. Introduction

Portal hypertension (PH) is a common clinical syndrome defined as the elevation of hepatic venous pressure gradient (HVPG) above 5 mm Hg. PH is caused by a combination of two simultaneous occurring hemodynamic processes: (1) increased intrahepatic resistance to passage of blood flow through the liver due to cirrhosis and (2) increased splanchnic blood flow secondary to vasodilatation within the splanchnic vascular bed. PH can be due to many different causes at prehepatic, intrahepatic, and posthepatic sites (Table 1). Cirrhosis of the liver accounts for approximately 90% of cases of PH in Western countries. 

The importance of PH is defined by the frequency and severity of its complications including variceal bleeding, spontaneous bacterial peritonitis, and hepatorenal syndrome, which represent the leading causes of death and of liver transplantation in patients with cirrhosis. PH is considered to be clinically significant when HVPG exceeds 10 to 12 mm Hg, since this is the threshold for the clinical complications of PH to appear [1]. Proper diagnosis and management of these complications are vital to improving quality of life and patients' survival. This paper will review the multisystemic manifestations of PH in cirrhosis.

## 2. Gastrointestinal Manifestations

### 2.1. Gastroesophageal (GE) Varices

Approximately 5–15% of cirrhotics per year develop varices, and it is estimated that the majority of patients with cirrhosis will develop GE varices over their lifetime. The presence of GE varices correlates with the severity of liver disease; while only 40% of child A patients have varices, they are present in 85% of child C patients (Table 2) [2].

Collaterals usually exist between the portal venous system and the systemic veins. The resistance in the portal vessels is normally lower than in the collateral circulation, and so blood flows from the systemic bed into the portal bed. However, when PH develops, the portal pressure is higher than systemic venous pressure, and this leads to reversal of flow in these collaterals. In addition, the collateral circulatory bed also develops through angiogenesis and the development of new blood vessels in an attempt to decompress the portal circulation [3]. The areas where major collaterals occur between the portal and systemic venous system are shown in Table 3. Unfortunately these collaterals are insufficient to decompress the PH, leading to complications including variceal bleeding.

GE area is the main site of formation of varices [4]. Esophageal varices (EV) form when the HVPG exceeds 10 mm Hg [5]. In the lower 2 to 3 cm of the esophagus, the varices in the submucosa are very superficial and thus have thinner wall. In addition, these varices do not communicate with the periesophageal veins and therefore cannot easily be decompressed. These are the reasons why EV bleeds only at this site.

Over the last decade, most practice guidelines recommend to screen known cirrhotics with endoscopy to look for GE varices. Varices should be suspected in all patients with stigmata of chronic liver disease such as spider nevi, jaundice, palmar erythema, splenomegaly, ascites, encephalopathy, and caput medusae. EV are graded as small (<5 mm) and large (>5 mm), where 5 mm is roughly the size of an open biopsy forceps [6].

The rate of progression of small EV to large is 8% per year [2]. Decompensated cirrhosis (child B or C), presence of red wale marks (defined as longitudinal dilated venules resembling whip marks on the variceal surface), and alcoholic cirrhosis at the time of baseline endoscopy are the main factors associated with the progression from small to large EV [2]. EV bleeding occurs at a yearly rate of 5%–15% [7]. The predictors of first bleeding include the size of varices, severity of cirrhosis (Child B or C), variceal pressure (>12 mm Hg), and the endoscopic presence of red wale marks [7, 8]. Although EV bleeding stops spontaneously in up to 40% of patients, and despite improvements in therapy over the last decade, the 6 weeks mortality rate is still ≥20% [9]. 

**Figure 1:**
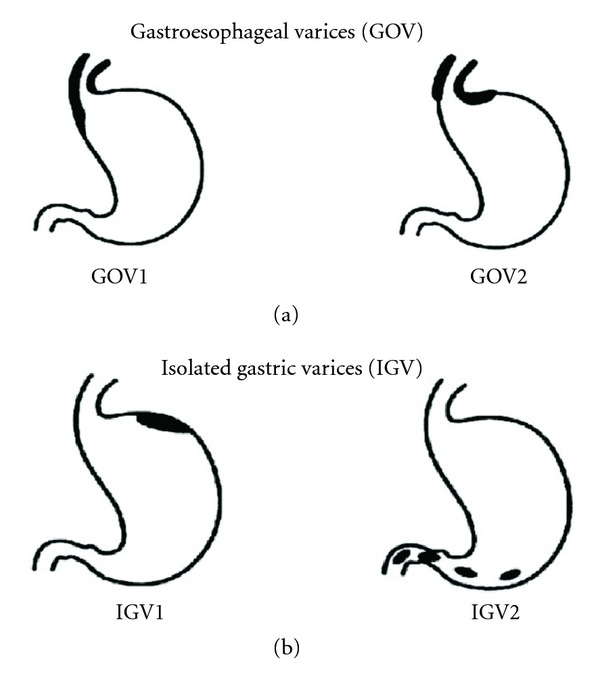
Sarin classification of gastric varices.

Gastroesophageal varices (GOV) are an extension of EV and are categorized based on Sarin's classification into 2 types (Figure 1). The most common are Type 1 (GOV1) varices, which extend along the lesser curvature. Type 2 GOV (GOV2) are those that extend along the fundus. They are longer and more tortuous than GOV1. Isolated gastric varices (IGV) occur in the absence of EV and are also classified into 2 types. Type 1 (IGV1) are located in the fundus and tend to be tortuous and complex, and type 2 (IVG2) are located in the body, antrum, or around the pylorus. When IGV1 is present, one must exclude splenic vein thrombosis. GV are less common than EV and are present in 5%–30% of patients with PH with a reported incidence of bleeding of about 25% in 2 years, with a higher bleeding incidence for fundal varices [10]. Predictors of GV bleeding include the size of fundal varices (large (>10 mm) > medium (5–10 mm) > small (<5 mm)), severity of cirrhosis (child class C>B>A), and endoscopic presence of variceal red spots (defined as localized reddish mucosal area or spots on the mucosal surface of a varix) [11].

### 2.2. Ectopic Varices (EcV)

EcV are best defined as large portosystemic venous collaterals occurring anywhere in the abdomen except for the GE region [12]. They are an unusual cause of GI bleeding, but account for up to 5% of all variceal bleeding [13]. Compared to GE varices, EcV are difficult to locate, occur at distal sites, and when identified, the choice of therapy is unclear, therefore representing a clinical challenge [12]. Furthermore, bleeding EcV may be associated with poor prognosis, with one study quoting mortality reaching 40% [14]. Different areas of EcV are the duodenum, jejunum, ileum, colon, rectum, peristomal, biliary tree, gallbladder, peritoneum, umbilicus, bare area of the liver, ovary, vagina, and testis [15, 16]. 

The prevalence of EcV varies in the literature and seems to be related to the etiology of the PH and the diagnostic modalities used [17]. In patients with PH due to cirrhosis, duodenal varices are seen in 40% of patients undergoing angiography [18]. Results of a survey for EcV conducted over 5 years in Japan identified 57 cases of duodenal varices; they were located in the duodenal bulb in 3.5%, the descending part in 82.5%, and the transverse part in 14.0% [15].

In contrast to duodenal varices, it appears that most cases of varices in other portions of the small bowel and colonic varices are seen in patients with cirrhosis who have previously undergone abdominal surgery [12]. Using advanced endoscopic technologies, particularly capsule endoscopy and enteroscopy, the prevalence of small bowel varices is estimated to be approximately 69% in patients with PH [19]. The prevalence of colonic varices and rectal varices has been found to be 34% to 46% [20, 21] and 10% to 40% [22], respectively, in patients with cirrhosis undergoing colonoscopy. It is important to differentiate rectal varices from hemorrhoids; rectal varices extend more than 4 cm above the anal verge, are dark blue in color, collapse with digital pressure, and do not prolapse into the proctoscope on examination, whereas hemorrhoids do not extend proximal to the dentate line, are purple in color, do not collapse with digital pressure, and often prolapse into the proctoscope [22, 23]. Stomal varices are a particularly common cause of EcV and can occur in patients with cirrhosis secondary to primary sclerosing cholangitis (PSC) [12]. 

In the west, because the prevalence of noncirrhotic PH is low, most bleeding EcV is usually associated with cirrhotic PH (6,8). Although EcV can occur at several sites, bleeding EcV are most commonly found in the duodenum and at sites of previous bowel surgery including stomas. 

In a review of 169 cases of bleeding EcV, 17% occurred in the duodenum, 17% in the jejunum or ileum, 14% in the colon, 8% in the rectum, and 9% in the peritoneum. In the review, 26% bled from stomal varices and a few from infrequent sites such as the ovary and vagina [24].

Portal biliopathy, which includes abnormalities (stricture and dilatation) of both extra and intrahepatic bile ducts and varices of the gallbladder, is associated with PH, particularly extrahepatic portal vein obstruction [25, 26]. They are also seen associated with cirrhosis, non-cirrhotic portal fibrosis, and congenital hepatic fibrosis [27]. While a majority of these patients are asymptomatic, some present with a raised alkaline phosphatase level, abdominal pain, fever, and cholangitis. Choledocholithiasis may develop as a complication and manifest as obstructive jaundice with or without cholangitis [26]. On cholangiography, bile-duct varices may be visualized as multiple, smooth, mural-filling defects with narrowing and irregularity resulting from compression of the portal vein and collateral vessels. They may mimic PSC or cholangiocarcinoma (pseudocholangiocarcinoma sign) [28]. 

### 2.3. Portal Hypertensive Intestinal Vasculopathies

Mucosal changes in the stomach in patients with PH include portal hypertensive gastropathy (PHG) and gastric vascular ectasia. PHG describes the endoscopicappearance of gastric mucosa with a characteristic mosaic, or snake-skin-like appearance with or without red spots. It is a common finding in patients with PH [29]. The prevalence of PHG parallels the severity of PH and it is considered mild when only a mosaic-like pattern is present and severe when superimposed discrete red spots are also seen. Bleeding (acute or chronic) from these lesions is relatively uncommon, and rarely severe [30]. Patients with chronic bleeding usually present with chroniciron deficiency anemia.

In gastric vascular ectasia, collection of ectatic vessels can be seen on endoscopy as red spots without a mosaic-like pattern [31]. When the aggregates are confined to the antrum of the stomach, the term gastric antral vascular ectasia (GAVE) is used, and if aggregates in the antrum are linear, the term watermelon stomach is used to describe the lesion. The prevalence of GAVE syndrome in cirrhosis is low [32] and can be endoscopically difficult to differentiate from severe PHG. Therefore, gastric biopsy may be required to differentiate them as histologically GAVE lesions are completely distinct from PHG (Table 4) [33]. 

Small bowel might also show mucosal changes related to PH, which is called portal hypertensive enteropathy (PHE). The diagnosis of PHE has been limited in the past due to the difficult access to the small bowel. With advanced endoscopic techniques such as capsule endoscopy and enteroscopy, PHE is now thought to be a frequent finding in patients with cirrhosis, perhaps as common as PHG, and may cause occult GI blood loss [34, 35].

Portal hypertensive colopathy (PHC) refers to mucosal edema, erythema, granularity, friability, and vascular lesions of the colon in PH. PHC may be confused with colitis [36, 37]. Although they are found in up to 70% of patients with PH and are more common in patients with EV and PHG, they rarely cause bleeding [38, 39].

### 2.4. Ascites and Spontaneous Bacterial Peritonitis (SBP)

Ascites is defined as the accumulation of free fluid in the peritoneal cavity. Cirrhotic PH is the most common cause of ascites, which accounts for approximately 75% patients with ascites. About 60% of patients with cirrhosis develop ascites during 10 years of observation [40]. The development of ascites is an important event in cirrhosis as the mortality is approximately 50% at 2 years without a liver transplantation [41]. The formation of ascites in cirrhosis is due to a combination of abnormalities in both renal function and portal and splanchnic circulation. The main pathogenic factor is sodium retention [42]. 

The main clinical symptom of patients with ascites is an increase in abdominal girth, often accompanied by lower-limb edema. In some cases, the accumulation of fluid is so severe that respiratory function and physical activity is impaired. In most cases, ascites develop insidiously over the course of several weeks. Patients must have approximately 1500 mL of fluid for ascites to be detected reliably by physical examination. Dyspnea in these patients can occur as a consequence of increasing abdominal distension and/or accompanying pleural effusions. Increased intra-abdominal pressure might favour the development of abdominal hernias (mainly umbilical) in patients with cirrhosis and longstanding ascites [43].

The current classification of ascites, as defined by the International Ascites Club, divides patients in three groups (Table 5) [44]. Patients with refractory ascites are those that do not respond to sodium restriction and high doses of diuretics or develop diuretic-induced side effects that preclude their use.

Ascites may not be clinically detectable when present in small volumes. In larger volumes, the classic findings of ascites are adistended abdomen with a fluid thrill or shifting dullness. Ascites must be differentiated from abdominal distension due to other causes such as obesity, pregnancy, gaseous distension of bowel, bladder distension, cysts, and tumours. Ultrasonography is used to confirm the presence of minimal ascites and guide diagnostic paracentesis.

Successful treatment depends on an accurate diagnosis of the cause of ascites. Paracentesis with analysis of ascitic fluid is the most rapid and cost-effective method of diagnosis. It should be done in patients with ascites of recent onset, cirrhotic patients with ascites admitted to hospital, or those with clinical deterioration. The most important analyses are cell count, fluid culture, and calculation of the serum: ascites albumin gradient (SAAG), which reflects differences in oncotic pressures and correlates with portal venous pressure. It SAAG is greater or equal to 1.1 g/dL (or 11 g/L), ascites is ascribed to PH with approximately 97% accuracy [45].

Patients with cirrhosis and ascites are also at risk of developing infections, particularly spontaneous bacterial peritonitis (SBP). SBP occurs in approximately 10% of hospitalized cirrhotic patients [46], with an associated mortality of 20–40% if untreated [47]. Many patients are asymptomatic, but clinical signs can include abdominal pain, fever, and diarrhea. The diagnosis of SBP is based on neutrophil count >250 cells/mm^3^ in the ascitic fluid.

## 3. Renal Manifestations

### 3.1. Hepatorenal Syndrome

Hepatorenal syndrome (HRS) is a common complication seen in patients with advanced cirrhosis and PH [48]. HRS can also be seen in other types of severe chronic liver disease, alcoholic hepatitis, or in acute liver failure. This syndrome generally predicts poor prognosis [48]. HRS has been defined in the literature as a reversible functional renal impairment in the absence of other causes of renal failure, tubular dysfunction, proteinuria, or morphological alterations in histological studies. Precise and accurate diagnostic criteria have been established in order to clearly define this syndrome (Table 6) [49]. The diagnosis remains one of exclusion. 

The reported incidence of HRS is approximately 10% among hospitalized patients with cirrhosis and ascites. The probability of occurrence of HRS in patients with cirrhosis is around 20% after 1 year and 40% after 5 years [50]. The pathogenesis of HRS is not completely understood, but is likely the result of an extreme underfilling of the peripheral arterial circulation secondary to arterial vasodilatation in the splanchnic circulation [51]. In addition, recent data indicates that a reduction in cardiac output also plays a significant role [52]. 

HRS-associated renal failure is seen in late stages of cirrhosis and is marked by severe oliguria, increased sodium and water retention, volume overload, hyperkalemia, and spontaneous dilutional hyponatremia. There are two main subtypes of HRS described [49]. Type 1 HRS is a rapidly progressive renal failure that is defined by doubling of serum creatinine >2.5 mg/dL (>221 *μ*moL/L) or a decrease of 50% in creatinine clearance (<20 mL/min) in less than 2 weeks. This form of HRS is usually precipitated by gastrointestinal bleeds, large volume paracenthesis, acute alcoholic hepatitis and SBP [53]. In addition to renal failure, patients with type 1 HRS present deterioration in the function of other organs, including the heart, brain, liver, and adrenal glands. The median survival of these patients without treatment is <2 weeks, and almost all of them die within 10 weeks after onset of HRS. Type 2 HRS is a moderate and stable renal failure with a serum creatinine of >1.5 mg/dL (>133 *μ*moL/L) that remains stable over a longer period and is characterized by diuretics resistant ascites [49, 54]. 

## 4. Neurological Manifestations

### 4.1. Hepatic Encephalopathy

Hepatic encephalopathy (HE) is defined as neurologic and psychiatric dysfunction in a patient with chronic liver disease. The exact mechanism leading to this dysfunction is still poorly understood, but multiple factors appear to play a role in its genesis. The liver normally metabolizes ammonia, produced by enteric bacteria [56] and enterocytes [57, 58]. In a patient with PH, ammonia bypasses the liver through portosystemic shunt and reaches the astrocytes in the brain. Within the astrocyte, ammonia is metabolized into glutamine, which acts as an osmole to attract water, thus causing cerebral edema. In addition, direct ammonia toxicity triggers nitrosative and oxidative stress, which lead to astrocyte mitochondrial dysfunction [59, 60]. Another important factor is the enhancement of gamma-aminobutyric acid (GABA-A) receptors through neuroinhibitory steroids (i.e., allopregnanolone) [61] and benzodiazepine. Benzodiazepine also contributes to astrocyte swelling through a specific receptor [62]. Finally, tryptophane byproducts indole and oxindole [63], manganese [64], inflammation, hyponatremia [65], and reduced acetylcholine through acetylcholinesterase activity [66] also contribute to cerebral dysfunction.

The clinical manifestations of HE can be subtle. Minimal hepatic encephalopathy (grade 0) (Table 7) can present with impaired driving ability [67], minimally impaired psychometric tests, decreased global functioning, and increased risk of falls [68]. In overt hepatic encephalopathy, diurnal sleep pattern changes will often precede neurologic symptoms. To add to the complexity, HE can be intermittent or persistent. 

The severity of presentation is usually classified using the West Haven criteria (Table 7). Grade 1 hepatic encephalopathy represents lack of awareness, anxiety or euphoria, and short attention span. Change of personality, lethargy, and inappropriate behavior can be seen in grade 2 encephalopathy. More advanced features include disorientation, stupor, confusion (grade 3), and can even reach coma (grade 4). Focal neurologic symptoms, including hemiplegia, may also be observed [69]. Physical examination may be normal, but typical signs include bradykinesia, asterixis, hyperactive deep tendon reflexes and even decerebrate posturing [55].

## 5. Pulmonary Manifestations

### 5.1. Hepatopulmonary Syndrome

Hepatopulmonary syndrome (HPS) is a triad of liver disease, pulmonary vascular ectasia and impaired oxygenation. HPS is defined in the literature as a widened alveolar-arterial oxygen difference (A-a gradient) in room air (>15 mm Hg or >20 mm Hg in patients > 64 years of age) with or without hypoxemia due to intrapulmonary vasodilatation in the presence of hepatic dysfunction [70, 71]. This syndrome occurs mostly in those with PH (with or without cirrhosis) and indicates poor prognosis and higher mortality. Estimates of the prevalence of HPS among patients with chronic liver disease range from 4 to 47%, depending upon the diagnostic criteria and methods used [71–73]. 

HPS results in hypoxemia through pulmonary microvascular vasodilatation and intrapulmonary arteriovenous shunting resulting in ventilation-perfusion mismatch [74], and can occur even with mild liver disease [75]. Clinically, patients with HPS complain of progressive dyspnea on exertion, at rest, or both. Severe hypoxemia (PaO_2_ < 60 mm Hg) is often seen and strongly suggests HPS [70, 71]. A classical finding in HPS is orthodeoxia defined as a decreased arterial oxygen tension by more than 4 mm Hg or arterial oxyhemoglobin desaturation by more than 5% with changing position from supine to standing. It is associated with platypnea defined as dyspnea worsened by upright position [70, 71]. Platypnea-orthodeoxia is caused by the worsening of diffusion-perfusion matching and increased shunting at the lung bases in the upright position. There are no hallmark signs on physical exam; however, cyanosis, clubbing, and cutaneous telangiectasia (spider nevi) are commonly noted. Furthermore, systemic arterioembolisation may cause stroke, cerebral hemorrhage, or brain abscess, and can present with neurological deficits. 

### 5.2. Portopulmonary Hypertension

Portopulmonary hypertension (PPH), a well-recognized complication of chronic liver disease, refers to pulmonary arterial hypertension (PAH) associated with PH when no alternative causes exist. It is defined by the presence of elevated pulmonary arterial pressure (mean pressure >25 mm Hg at rest and 30 mm Hg on exertion) elevated pulmonary vascular resistance (>240 dyne s^−1^ cm^−5^) in the presence of a pulmonary capillary wedge pressure <15 mm Hg [76]. 

The prevalence of PPH depends on the patient population studies and severity of the liver disease, 0.7–2% and 3.5–16.1% in cirrhotics and patients undergoing liver transplantation, respectively. The development of PPH is independent of the cause of PH, and it is often seen in cirrhosis. It is however, also described in those with PH due to nonhepatic pathologies such as portal venous thrombosis [71, 77]. PH seems to be the driving force of PAH. The pathogenesis of PPH is not completely understood; however, several theories have been offered. The most widely accepted theory is that a humoral vasoactive substances (e.g., serotonin, endothelin-1, interleukin-1, thromboxane B2, and secretin), normally metabolized by the liver, is able to reach the pulmonary circulation via portosystemic shunts, resulting in PPH [71, 78, 79].

Clinically, most patients with PPH present with evidence of both PAH and PH. Typically manifestations of PH precede those of PAH. The most common presenting symptom is progressive dyspnea on exertion [80] and less frequently fatigue, palpitations, syncope, hemoptysis, orthopnea, and chest pain. On physical exam, classical features include edema, an accentuated P2 and a systolic murmur, indicating tricuspid regurgitation [71, 77, 80]. In severe cases, signs and symptoms of right-heart failure can be noted. 

### 5.3. Hepatic Hydrothorax

Hepatic hydrothorax is an uncommon complication of end-stage liver disease. It is defined as a pleural effusion greater than 500 mL in patients with cirrhosis in absence of primary cardiac, pulmonary, or pleural disease [81]. The underlying pathogenesis of hepatic hydrothorax is incompletely understood. Patients with cirrhosis and PH have abnormal extracellular fluid volume regulation resulting in passage of ascites from the peritoneal space to the pleural cavity via diaphragmatic defects generally in the tendinous portion of the diaphragm [82]. Negative intrathoracic pressure during inspiration pulls the fluid from the intra-abdominal cavity into the pleural cavity. Hydrothorax develops when the pleural absorptive capacity is surpassed, leading to accumulation of fluid in the pleural space. Multiple studies have shown the passage of fluid from the intra-abdominal space to the pleural space via 99mTc-human albumin or 99mTc-sulphur colloid [81]. 

Clinical manifestations of hepatic hydrothorax include shortness of breath, cough, hypoxemia, and chest discomfort [81]. Ascites may not always be present. Hepatic hydrothorax should always be suspected in patients with cirrhosis or PH and undiagnosed pleural effusion, regardless of the presence of ascites. Serious complications include acute tension hydrothorax with dyspnoea and hypotension [83] and spontaneous bacterial empyema [84].

## 6. Other Organs Manifestations

### 6.1. Cirrhotic Cardiomyopathy

Cirrhotic cardiomyopathy is defined as a chronic cardiac dysfunction in patients with cirrhosis. It occurs in up to 50% of patients with advanced cirrhosis. It is characterized by impaired contractile response and/or altered diastolic relaxation in the absence of other cardiac diseases. The pathophysiology of this condition is complex, and seemingly related to PH and cirrhosis. In advanced liver disease, splanchnic vasodilatation leads to a resting hyperdynamic state [85]. Plasma volume expands, leading to a relative central volume decrease [86]. In cirrhosis, the arterial vessel wall thickness and tone decreases, leading to reduced arterial compliance [87, 88]. Autonomic dysfunction may also contribute to blunted cardiac response [89]. Ultimately, these factors lead to systolic and diastolic dysfunction. 

Symptoms associated with cirrhotic cardiomyopathy include dyspnea with exertion, impaired exercise capacity, paroxysmal nocturnal dyspnea, peripheral edema, and orthopnea. Less-frequent presentations include long QT on electrocardiography, arrhythmia, and sudden death [90].

### 6.2. Hepatic Osteodystrophy

Hepatic osteodystrophy is defined as bone disease (osteomalacia, osteoporosis, and osteopenia) associated with liver disease. Osteomalacia and osteoporosis are frequently seen in cirrhotic patients and can predispose to pathologic fractures. The pathophysiology of osteoporosis in liver disease is relatively complex. The leading hypothesis suggests that it is related to the uncoupling of osteoblastic and osteoclastic activity. Osteoclastogenic proinflammatory cytokines (interleukin 1(Il-1) and tumor necrosis factor *α* (TNF*α*)) are increased in hepatic fibrosis. Moreover, TNF*α* is increased in a rat model of PH [91]. Decreased osteoblastic activity has also been linked with insulin-like growth factor 1 in a rat model (IGF-1). Increasing IGF-1 levels are associated with liver disease severity [92]. Finally, vitamin K mediates the carboxylation of glutamyl residues on osteocalcin, stimulating osteoclastic activity [93]. 

Patients with osteoporosis are usually asymptomatic. They may present with pain following a nontraumatic fracture of the axial skeleton or bone deformity, including pronounced cervical kyphosis. Osteomalacia presentation is similar and includes proximal muscle weakness [94].

### 6.3. Hypersplenism

Hypersplenism is a common complication of massive congestive splenomegaly in patients with cirrhosis and PH. In this condition, splenomegaly is associated with thrombocytopenia, leucopenia, or anemia or a combination of any the three [95, 96]. Severe hypersplenism is present in about 1/3 of patients with cirrhosis being assessed for liver transplantation. Most patients have no symptoms related to hypersplenism, however severe thrombocytopenia may increase the risk of bleeding, especially after invasive procedures. 

## 7. Conclusion

Portal hypertension secondary to cirrhosis has multisystem effects and complications. Once a patient develops such complications, they are considered to have decompensated disease with the high morbidity and mortality. The quality of life and survival of patients with cirrhosis can be improved by the prevention and treatment of these complications.

## Figures and Tables

**Table 1:** Causes of portal hypertension (PH).

Prehepatic PH (normal wedged hepatic venous pressure (WHVP) and free hepatic venous pressure (FHVP) with normal hepatic venous pressure gradient (HVPG))	
Portal vein thrombosis	
Splenic vein thrombosis	
Congestive splenomegaly (Banti's syndrome)	
Arteriovenous fistula	
Hepatic PH (increased WHVP, normal FHVP, and increased HVPG)	
Presinusoidal	
Schistosomiasis	
Congenital hepatic fibrosis	
Sinusoidal	
Cirrhosis—many causes	
Alcoholic hepatitis	
Nodular regenerative hyperplasia	
Polycystic liver disease	
Postsinusoidal	
Sinusoidal obstructive syndrome	
Budd-Chiari syndrome	
Posthepatic PH (increased WHVP and FHVP and normal HVPG)	
Inferior vena cava webs, thrombosis	
Cardiac causes (restrictive cardiomyopathy, constrictive pericarditis, and congestive heart failure)	
Pulmonary hypertension	

**Table 2:** Child-Pugh-Turcotte (CPT) Classification of the Severity of Cirrhosis.

Parameter	Points assigned		
	1	2	3
Ascites	None	Mild/Moderate	Tense
Hepatic encephalopathy	None	Grade 1-2	Grade 3-4
Bilirubin micromol/L (mg/dL)	<34.2 (<2)	34.2–51.3 (2-3)	>51.3 (>3)
Albumin g/L (g/dL)	>35 (>3.5)	28–35 (2.8–3.5)	<28 (<2.8)
PT (Sec over control) or INR	<4	4–6	>6
	<1.7	1.7–2.3	>2.3
CPT classification Child A: 5-6 points Child B: 7-9 points Child C: 10–15 points			

**Table 3:** Location and blood vessels of collaterals between the portal and systemic venous circulations.

Location	Postal circulation	Systemic circulation
Gastroesophageal junction	Short gastric and left gastric (coronary) veins	Azygos vein
Rectum	Superior hemorrhoidal veins	Middle and inferior hemorrhoidal veins
Umbilical (caput medusa)	Left portal via a recannulated umbilical vein	Epigastric venous plexus of the abdominal wall
Retroperitoneum	Mesentric veins	Intercostal, phrenic, lumbar, and renal veins

**Table 4:** Comparison of portal hypertensive gastropathy (PHG) and gastric antral vascular ectasia (GAVE).

FEATURE	PHG	GAVE
Relation with PH	Causal	Coincidental
Distribution in stomach	Mainly proximal	Mainly distal
Mosaic pattern	Present	Absent
Red color signs	Present	Present
Pathology		
Thrombi	−	+++
Spindle cell proliferation	+	++
Fibrohyalinosis	−	+++
Treatment	*β*-adrenergic blockers TIPS/shunt surgery	Endoscopic Antrectomy and Billroth I Liver transplantation

PH: portal hypertension, TIPS: transjugular intrahepatic portosystemic shunt.

**Table 5:** International ascites club grading system for ascites.

Grade of ascites	Definition
Grade 1 ascites	Mild ascites only detectable by ultrasound
Grade 2 ascites	Moderate ascites evident by moderate symmetrical distension of abdomen
Grade 3 ascites	Large or gross ascites with marked abdominal distension

**Table 6:** Revised diagnostic criteria of Hepatorenal syndrome.

(i) Chronic or acute liver disease with advanced liver failure and portal hypertension	
(ii) Plasma creatinine concentration > 1.5 mg/dL (133 micromol/L)	
(iii) The absence of other apparent cause: shock, ongoing bacterial infection, volume depletion, current or recent use of nephrotoxic drugs	
(iv) Lack of improvement in renal function after volume expansion with intravenous albumin (1 g/kg of body weight per day up to 100 g/day) for at least two days and withdrawal of diuretics	
(v) Absence of parenchymal kidney disease as indicated by proteinuria >500 mg/day, microhematuria (>50 red blood cells per high power field) or ultrasonographic evidence of obstructive uropathy or renal parenchymal disease	

**Table 7:** West Haven Criteria of Severity of Hepatic Encephalopathy (Adapted with permission [55]).

Grade 1	Trivial lack of awareness Euphoria or anxiety Shortened attention span Impaired performance of addition

Grade 2	Lethargy or apathy Minimal disorientation for time and place Subtle personality change Inappropriate behavior Impaired performance of subtraction

Grade 3	Somnolence to semistupor, but responsive to verbal stimuli Confusion Gross disorientation

Grade 4	Coma (unresponsive to verbal or noxious stimuli)
